# An exploratory plasma-based functional assay for phenotypic characterization of fibrinolysis in dysfibrinogenemia

**DOI:** 10.1016/j.rpth.2026.103426

**Published:** 2026-03-25

**Authors:** Atsuo Suzuki, Shuichi Okamoto, Nobuaki Suzuki, Shogo Tamura, Takeshi Kanematsu, Masashi Tomita, Tetsuhito Kojima, Tadashi Matsushita

**Affiliations:** 1 Department of Clinical Laboratory, Nagoya University Hospital, Nagoya, Japan; 2 Division of Cellular and Genetic Sciences, Department of Integrated Health Sciences, Nagoya University Graduate School of Medicine, Nagoya, Japan; 3 Department of Transfusion Medicine, Nagoya University Hospital, Nagoya, Japan; 4 Department of Clinical Laboratory Science, Faculty of Health Sciences, Hokkaido University, Sapporo, Japan; 5 Aichi Health Promotion Foundation, Nagoya, Japan

**Keywords:** dysfibrinogenemia, fibrinogen, fibrinolysis, medical laboratory science, thrombosis

## Abstract

**Background:**

Congenital dysfibrinogenemia exhibits heterogeneous clinical phenotypes, ranging from bleeding to thrombosis. However, conventional fibrinogen assays primarily evaluate clot formation alone. Genetic analyses are informative but do not fully explain phenotypic diversity, particularly with respect to clot formation–lysis behavior.

**Objectives:**

This study developed a novel plasma-based functional assay for exploratory characterization of integrated clot formation–lysis kinetics in congenital dysfibrinogenemia.

**Methods:**

The Clauss fibrinogen assay was modified by supplementing recombinant tissue-type plasminogen activator and Lys-plasminogen to enable monitoring of the fibrinolysis phase following clot formation, termed the CLySis assay. Clot-fibrinolysis waveform analysis defined the time to fibrinolysis (*T*^lysis^) and maximum fibrinolysis velocity. Normal reference intervals were established, and the assay was applied to plasma samples from 22 patients with congenital fibrinogen disorders (19 dysfibrinogenemia and 3 hypofibrinogenemia), including carriers of variants previously reported in patients with thrombosis.

**Results:**

The CLySis assay enabled stable monitoring of clot formation and subsequent fibrinolysis. The *T*^lysis^ was largely independent of plasma fibrinogen concentration, whereas maximum fibrinolysis velocity was influenced by fibrinogen levels. Among 5 variants previously reported in patients with thrombosis, FGA p.Arg35Cys, FGG p.Arg301Cys, and FGG p.Asp344Gly showed markedly prolonged *T*^lysis^ compared with the normal reference range, indicating altered clot formation–lysis kinetics. By contrast, FGB p.Arg74Cys and FGG p.Arg301His showed *T*^lysis^ values within the normal range. Variants without reported thrombotic association also demonstrated normal clot–lysis profiles.

**Conclusion:**

The CLySis assay provides a functional, genotype-independent approach for exploratory characterization of clot–lysis behavior in congenital dysfibrinogenemia. This assay may support future phenotypic analyses of dysfibrinogenemia, complementing conventional genetic evaluation.

## Essentials

• Dysfibrinogenemia shows heterogeneous clinical phenotypes.
• We modified the Clauss fibrinogen assay to monitor clot formation and fibrinolysis.
• Certain variants showed prolonged clot formation–lysis kinetics.
• This novel approach may improve understanding of dysfibrinogen properties.

## 1 Introduction

Congenital fibrinogen disorders (CFDs) are classified into 2 defect groups: quantitative and qualitative. A more detailed subclassification has been proposed by the Scientific and Standardization Committee of the International Society on Thrombosis and Haemostasis (ISTH) to reflect the wide spectrum of abnormalities observed in these disorders [1]. Among CFDs, the qualitative abnormality dysfibrinogenemia is clinically heterogeneous and manifests as bleeding and thrombosis, but in some cases, the condition remains asymptomatic [[2], [3], [4]]. Thrombotic events are generally considered less common than bleeding in patients with dysfibrinogenemia [3,[5], [6], [7], [8], [9]], but the reported frequency varies across studies, and the true incidence has not been firmly established.

Thrombosis in dysfibrinogenemia appears paradoxical, and several mechanisms have been proposed to explain its development as follows: (1) defective thrombin binding by abnormal fibrinogen (Fg), leading to elevated levels of circulating thrombin; and (2) formation of fibrin clots that show impaired binding of plasminogen (Pg) or tissue-type Pg activator (tPA), resulting in resistance to fibrinolysis. In some cases, thrombotic phenotypes of CFDs can be partially predicted by variants in Fg genes (*FGA*, *FGB*, and *FGG*). Indeed, genotype–phenotype associations have been reported for certain variants [5,10]. However, even carriers of the same mutation can exhibit divergent clinical outcomes. Larger cohort studies have shown that some patients harboring mutations associated with thrombosis instead exhibit bleeding tendencies, suggesting that clinical phenotypes are affected by individual variability [[4], [5], [6], [7], [8]]. Therefore, genotype analysis alone cannot reliably predict the clinical manifestations of dysfibrinogenemia [11,12], and neither the level of functional fibrinogen (Fg:C) nor the ratio of Fg:C to antigenic fibrinogen (Fg:Ag) have been associated with thrombotic risk [3]. Thus, phenotypic diagnosis remains a major challenge.

The severity of bleeding with quantitative defects is correlated with the level of Fg:C, but this relationship is not observed with qualitative defects [8]. Although several specialized functional assays have been developed [4,13], they are technically demanding and not standardized and thus not suitable for routine phenotypic evaluation. We recently developed a screening test based on the Clauss Fg assay—the most widely used method for assessing plasma Fg—combined with clot waveform analysis (Clauss-CWA), which enables the differentiation of qualitative vs quantitative Fg disorders [14,15]. The Clauss-CWA not only provides the Fg:C but also estimates the antigenic fibrinogen level (Fg:eAg). The latter is derived from the maximum coagulation velocity, which is closely correlated with the plasma Fg:eAg obtained from clot waveform data. Thus, both functional and antigenic properties of Fg can be inferred from a single measurement [15]. In addition, this method mathematically models the clotting reaction from the clot waveform, and CWA can also be extended to the monitoring of fibrinolysis after clot formation. The fibrinolysis phase can be triggered by various factors, such as tPA, and can be tracked as a clot-fibrinolysis waveform [16].

The established Clauss-CWA primarily monitors the conversion of Fg to fibrin. However, the Clauss-CWA alone is insufficient for phenotypic characterization of dysfibrinogenemia, as it focuses exclusively on the clotting phase, particularly Fg-to-fibrin conversion, despite the fact that hemostasis involves sequential processes of clot formation and subsequent fibrinolysis. Extending Clauss-CWA to include the fibrinolytic phase may therefore provide additional information on fibrin properties related to clot formation–lysis kinetics in patient plasma. Because certain dysfibrinogenemia variants have been reported to exhibit altered fibrinolytic behavior, monitoring this phase may help to functionally characterize differences in clot formation–lysis profiles among dysfibrinogenemia. Accordingly, the aim of the present study was to establish a novel laboratory assay to characterize integrated clot formation and fibrinolysis kinetics in dysfibrinogenemia by modifying the Clauss-CWA assay.

## 2 Methods

### 2.1 Plasma samples

This study was approved by the Nagoya University Hospital Ethics Committee (approval no. 2022-0317), and conducted in accordance with the ethical standards established by the 1964 Declaration of Helsinki and later amendments. Race/ethnicity data were not formally collected; however, all participants were of Japanese origin, reflecting the local patient population. Blood samples were collected from healthy donors and patients previously diagnosed with CFD in polypropylene tubes containing 0.109 mol/L sodium citrate. Platelet-poor plasma was prepared by centrifugation at 2000 × *g* for 10 minutes and stored at −80 °C until use. CRYOcheck Normal Donor Set (Precision BioLogic Inc) was used as a source of normal reference plasma. Plasma α_2_-antiplasmin (α_2_AP) activity was measured using a chromogenic assay with Revohem Antiplasmin reagent (Sysmex Corp) on a CN-6000 autoanalyzer (Sysmex). Pooled normal plasma (PNP) was prepared using normal plasma samples from health donors (>25 individuals). Samples of immunoabsorbed frozen plasma deficient in Fg, Pg, α_2_AP, Pg activator inhibitor (PAI)-1, and thrombin-activatable fibrinolysis inhibitor (TAFI) were purchased from Affinity Biologicals.

### 2.2 Fibrinogen assays

Fg:C was measured using the Clauss Fg assay with Thrombocheck FibL reagent (Sysmex) on a CN-6000 autoanalyzer (Sysmex) [15]. Fg:Ag levels were measured using a latex immunoturbidimetric assay [14]. Both assays were calibrated using same plasma-based calibrator, Coagtrol N (Sysmex), a traceable WHO Third International Standard for plasma Fg. An Fg:C-to-Fg:Ag ratio of 0.55 was used as the cutoff to distinguish qualitative defects from quantitative defects [15].

### 2.3 Clauss fibrinogen assay–based clot-fibrinolysis waveform analysis

**Figure 1:**
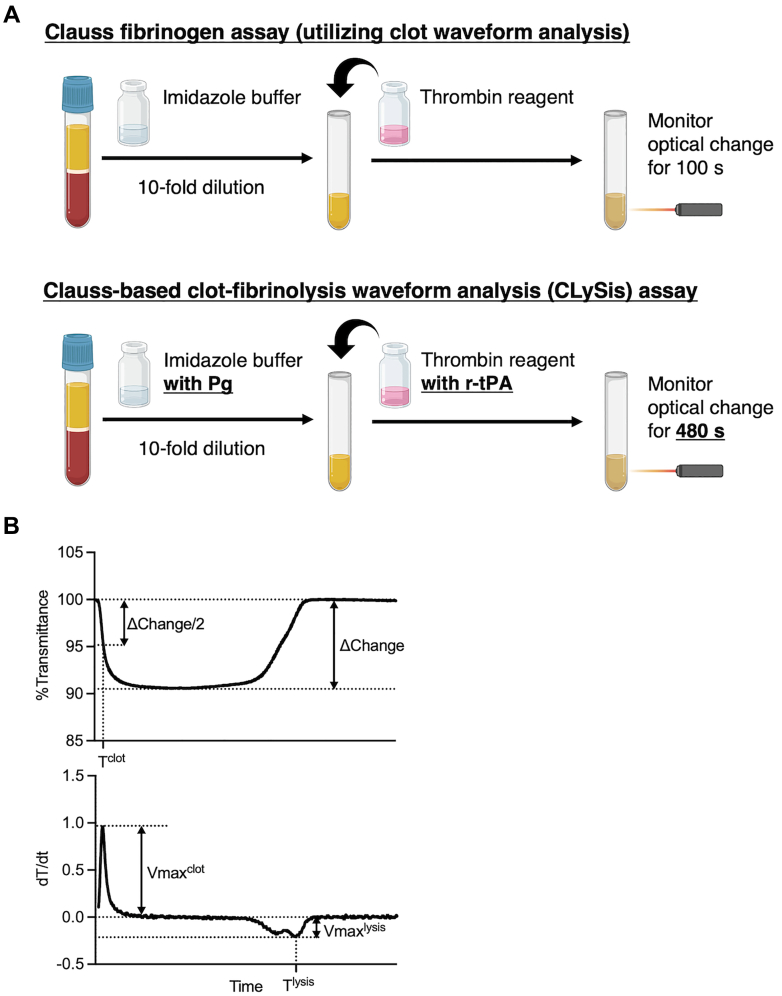
Schematic illustration of the CLySis assay procedure and representative clot-fibrinolysis waveform with the first-derivative curve. (A) In the original Clauss fibrinogen (Fg) assay, plasma samples were diluted 10-fold and mixed with thrombin reagent before monitoring optical changes (upper illustration). In Clauss Fg assay–based clot-fibrinolysis waveform analysis (CLySis assay), citrated plasma is diluted 10-fold with an imidazole buffer supplemented with Lys-plasminogen (Pg). The diluted plasma is then mixed with thrombin reagent containing recombinant tissue-type Pg activator (r-tPA), and optical transmittance is monitored at 405 nm for 480 seconds (lower illustration). (B) A representative clot-fibrinolysis waveform and first-derivative curve are shown together with the corresponding clotting and fibrinolysis parameters. Clotting time (*T*^clot^) is defined as the time point at which 50% of the total transmittance change (ΔChange/2) is reached. Maximum clotting velocity (*V*_max_^clot^) is indicated by the positive peak height, and maximum fibrinolysis velocity (*V*_max_^lysis^) is defined as the negative peak height of the first-derivative curve. Time of fibrinolysis (*T*^lysis^) is determined as the time corresponding to *V*_max_^lysis^.

The Clauss fibrinogen assay–based clot-fibrinolysis waveform analysis, namely CLySis assay, was developed by supplementing the standard reagents used for the Clauss Fg assay with fibrinolysis-triggering components. Figure 1A shows a comparison of reagents and protocols between the Clauss fibrinogen assay (Clauss-CWA) and the CLySis assay. Test plasma was diluted 1:9 with imidazole buffer (Sysmex) containing human Lys-Pg or Glu-Pg (Enzyme Research Laboratories). After incubation at 37 °C for 190 seconds, thrombin reagent (Thrombocheck FibL) supplemented with recombinant tissue-type Pg activator (r-tPA; arteplase; Activacin; Kyowa Kirin) was added. As the r-tPA solution was added at 1/1000th of the total volume, the dilution effect was considered negligible. Clot formation and subsequent fibrinolysis phases were monitored by measuring the light transmittance using a CS-5100 autoanalyzer (Sysmex).

Figure 1B shows the representative clot-fibrinolysis waveform and its derivative curve. Clotting time (*T*^clot^) was defined as the time point at which 50% of the total change in transmittance (ΔChange) had occurred. Reaction velocity was quantified from the first derivative of the clot-fibrinolysis waveform. Maximum velocity (*V*_max_) was defined as the peak value of the first-derivative curve; *V*_max_ during clot formation was denoted as *V*_max_^clot^, and *V*_max_ during fibrinolysis was denoted as *V*_max_^lysis^. Fibrinolysis time (*T*^lysis^) was defined as the time to reach *V*_max_^lysis^, reflecting integrated clot formation–lysis kinetics rather than isolated fibrinolytic activity.

The analytical precision of the CLySis assay was evaluated using Control Plasma N and P (Siemens Healthineers). Each control was measured in 5 replicates on 5 independent days. The coefficients of variation for *T*^lysis^ were 3.4% for Control Plasma N and 3.6% for Control Plasma P, respectively, and those for *V*_max_^lysis^ were 3.7% and 5.5%, respectively.

### 2.4 Purification of plasminogen-free Fg

Pg-free Fg was purified from citrated plasma following the procedure originally described by Walker and Catlin [17]. Fibrinogen precipitate was finally dissolved in 20 mM HEPES-buffered saline (pH 7.4) and dialyzed extensively against 20 mM HEPES-buffered saline (pH 7.4) using a Slide-A-Lyzer MINI dialysis device (20K MWCO; Thermo Fisher Scientific). The absence of Pg in the purified Fg was confirmed by demonstrating the absence of fibrinolysis following addition of tPA and thrombin. The concentration of purified Fg solution was determined as Fg:Ag measured using an immunoturbidimetric assay. The effectiveness of purification was confirmed via comparison with commercial Pg-depleted purified Fg (FIB1; Enzyme Research Laboratories).

### 2.5 Plasmin generation assay

Colorimetric plasmin generation (PG) assays were performed according to previous studies [18,19]. Pg-depleted purified Fg (50 μg/mL) was mixed with α-thrombin (0.5 U/mL; Enzyme Research Laboratories), r-tPA (1 nM), Glu-Pg (0.25 μM), chromogenic substrate S-2251 (0.5 mM; Chromogenix), and CaCl_2_ (1 mM). The absorbance of the resulting mixture was measured at 405 nm with a reference absorbance of 650 nm at 37 °C every 1 minute for 150 min using a BioTek Citation 5 imaging multimode reader (Agilent Technologies).

### 2.6 Clot lysis assay

Clot lysis assays were performed according to previous studies using purified Fg derived from patients with CFD [[18], [19], [20]]. Pg-depleted purified Fg (0.5 mg/mL) was clotted in a microtiter plate by addition of α-thrombin at a final concentration of 1 U/mL. After incubation at 37 °C for 1 hour, a fibrinolysis-triggering mixture containing Glu-Pg (0.29 μM) and r-tPA (1 nM) was layered onto the formed clot, and the absorbance was monitored at 340 nm every 1 minute for 150 minutes at 37 °C using a BioTek Citation 5 imaging multimode reader. Clot lysis percentage was calculated as described elsewhere [20]: %Lysis = 100 − (A340 × 100)/maximum A340.

## 3 Results

### 3.1 Development of the CLySis assay

**Figure 2:**
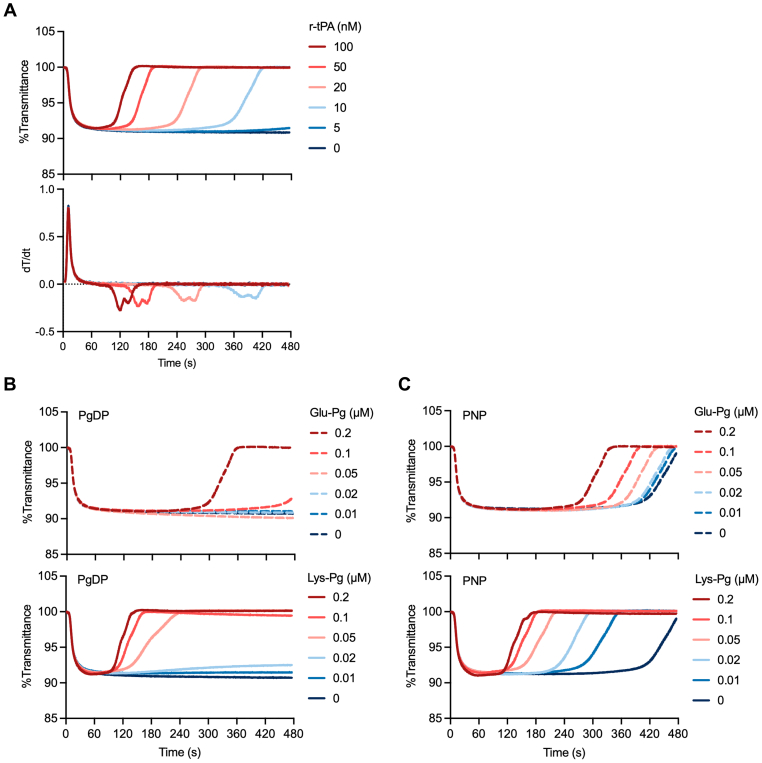
Optimization of the Clauss fibrinogen assay–based clot-fibrinolysis waveform analysis (CLySis) assay system. (A) Dose-dependent changes in clot-fibrinolysis waveforms and first-derivative curves. Fibrinolysis was induced by supplementing thrombin reagent alone with recombinant tissue-type plasminogen (Pg) activator (r-tPA) at 5 to 100 nM. (B, C) Imidazole buffer was supplemented with Glu- or Lys-Pg at final concentrations of 0.01 to 0.2 μM (in 10-fold–diluted plasma), and fibrinolysis was triggered by 10 nM r-tPA. The efficacy of Pg supplementation was assessed using Pg-deficient plasma (Pg-DP) (B) or pooled normal plasma (PNP) (C).

To monitor the fibrinolytic phase in the Clauss Fg assay, tPA was added to the thrombin reagent at final concentrations ranging from 5 to 100 nM to determine the optimal concentration for evaluating fibrinolysis. Fibrinolysis was detected as a change in the transmittance of light returning to the baseline in a dose-dependent manner, with higher concentrations of tPA leading to earlier onset of lysis. Almost no fibrinolysis was observed over the 480-second measurement period at the lowest tPA concentration of 5 nM (Figure 2A). The changes in *T*^lysis^ and *V*_max_^lysis^ were dose dependent, but the clotting phases, represented by *T*^clot^ and *V*_max_^clot^, were not affected by addition of tPA, even at the highest concentration of 100 nM. To distinguish the clotting and fibrinolytic phases and accurately assess fibrinolysis, the tPA concentration was fixed at 10 nM for subsequent experiments.

We then analyzed normal plasma samples (*n* = 50) using the tPA-supplemented Clauss Fg assay. However, *T*^lysis^ and *V*_max_^lysis^ values were highly variable, and the ranges (301.6-472.7 seconds for *T*^lysis^; −0.025 to −0.249 for *V*_max_^lysis^) were too broad to establish a normal reference. In this assay, fibrinolysis depends in part on the concentration of Pg in plasma; however, as the plasma samples were diluted 10-fold, the lower Pg concentration may have contributed to the instability of the test’s performance. Therefore, the sample diluent was externally supplemented with Pg to stabilize the assay. We compared 2 types of modified Pg—Glu- and Lys-Pg, to identify the most effective type (Supplementary Table S1). First, Pg-deficient plasma (DP) was tested to evaluate the effects of supplementation with Glu- or Lys-Pg (Figure 2B). Only the highest concentration of Glu-Pg (0.2 μM, ∼one-tenth of the normal plasma level) produced measurable fibrinolysis, whereas lower concentrations (≤0.1 μM) failed to induce lysis within the 480-second observation period. When using Lys-Pg, fibrinolysis was observed at concentrations of 0.05 to 0.2 μM, but not at ≤0.02 μM.

The effects of supplementation with Glu- or Lys-Pg were also assessed using the same series of concentrations with PNP as the test sample (Figure 2C). As PNP contains endogenous Pg, fibrinolysis occurred even without Glu- or Lys-Pg supplementation, and no significant synergistic effects were observed at Glu-Pg concentrations up to 0.02 μM. Addition of >0.05 μM Glu-Pg resulted in a dose-dependent shortening of *T*^lysis^, but the value was similar to that in Pg-DP, even when using 0.2 μM Glu-Pg. By contrast, supplementation with Lys-Pg resulted in a dose-dependent shortening of *T*^lysis^ from the lowest Lys-Pg concentration (0.01 μM). This shortening began to plateau at a Lys-Pg concentration of 0.1 μM. Values of both *V*_max_^lysis^ and *T*^lysis^ changed in a dose-dependent manner, indicating that higher Pg concentrations increase the rate of fibrinolysis. These results suggest that the concentration of endogenous Glu-Pg in test plasma samples is insufficient and that Lys-Pg is more effective than Glu-Pg at lower concentrations. Based on these findings, all subsequent experiments included 0.05 μM Lys-Pg (final concentration in the diluted plasma) and 10 nM r-tPA.

### 3.2 Impact of fibrinolysis-related factors in CLySis assay

**Figure 3:**
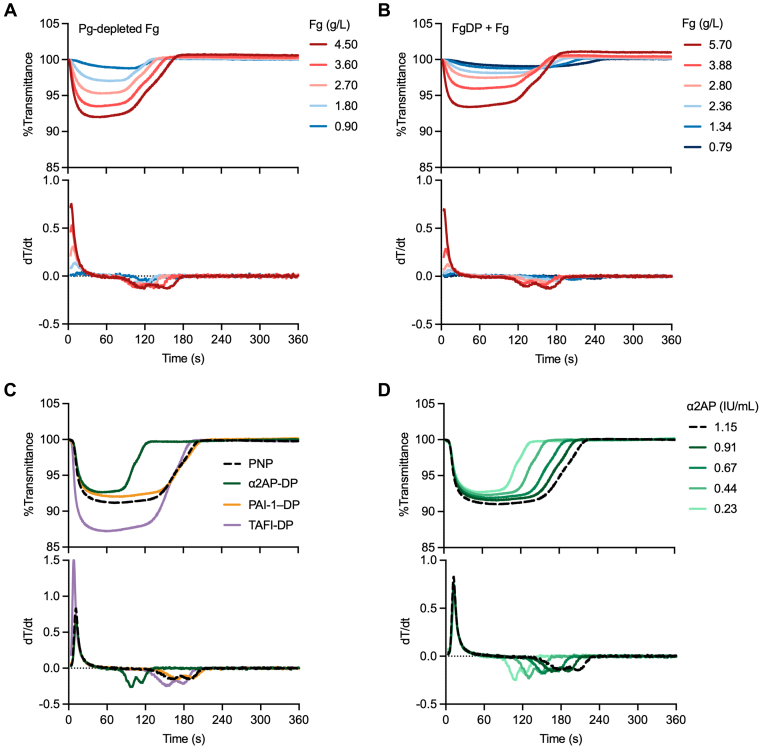
Effect of fibrinogen (Fg) and antifibrinolytic factor levels on the Clauss Fg assay–based clot-fibrinolysis waveform analysis (CLySis) assay. (A, B) Effects of changes in Fg level on the CLySis assay were investigated using Pg-depleted purified Fg (A) and purified Fg-spiked Fg-deficient plasma (FgDP) (B). The assigned Fg levels were determined using the Clauss Fg assay. (C) CLySis assay results for plasma α_2_-antiplasmin (α_2_AP)-, plasminogen (Pg) activator inhibitor (PAI)-1–, and thrombin-activatable fibrinolysis inhibitor (TAFI)-deficient plasma. Dashed line indicates the normal reference waveform obtained from pooled normal plasma (PNP). (D) Changes in CLySis assay results depending on the α_2_AP level in test plasma. α_2_AP-deficient plasma was mixed with PNP (dashed line) at various ratios, and the actual α_2_AP activity was measured using a chromogenic assay.

We first investigated how Fg levels in test plasma affect assay results. Two types of samples were used as follows: (1) purified Fg alone and (2) reconstituted plasma prepared by adding purified Fg to Fg-DP. The CLySis assay was performed using Pg-depleted, purified Fg at antigenic Fg levels ranging from 0.90 to 4.50 g/L. Both *T*^lysis^ and *V*_max_^lysis^ decreased in a concentration-dependent manner, with decreasing Fg level (Figure 3A, Supplementary Table S2). Dose-dependent changes were also observed with reconstituted plasma samples; however, *T*^lysis^ was prolonged at lower Fg concentrations (≤1.34 g/L) (Figure 3B, Supplementary Table S3). These differences between sample types suggest that plasma-derived components affect fibrinolysis and that certain fibrinolysis-related factors may affect CLySis assay results.

The effects of fibrinolysis-related factors on the CLySis assay were evaluated using α_2_AP-, PAI-1–, or TAFI-DP. As shown in Figure 3C, the results obtained with PAI-1–DP and TAFI-DP were almost identical to those obtained with PNP, whereas α_2_AP-DP induced a marked shortening of *T*^lysis^ and an increase in *V*_max_^lysis^. These changes gradually normalized when α_2_AP-DP was mixed with PNP at various ratios, corresponding to actual α_2_AP activities ranging from 0.23 to 1.15 IU/mL (Figure 3D). Taken together, these results indicate that plasma α_2_AP activity has a significant impact on the fibrinolytic phase in the CLySis assay and that assessing the plasma α_2_AP level may be important for accurate interpretation of assay results.

### 3.3 Investigation of dysfibrinogenemia

**Figure 4:**
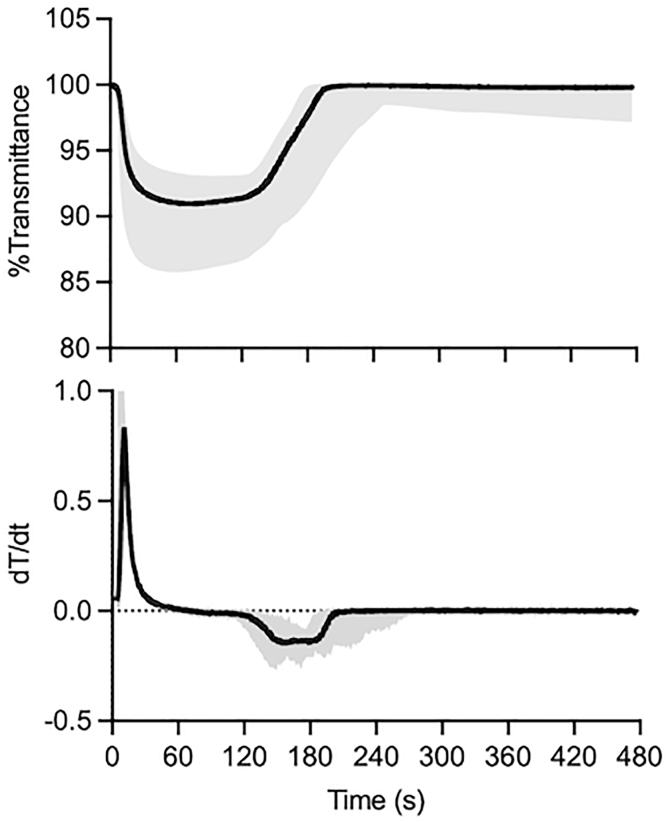
Normal reference ranges of the Clauss fibrinogen assay–based clot-fibrinolysis waveform analysis (CLySis) assay. Normal reference ranges were established using plasma samples from 50 healthy individuals. Solid lines represent the median value, and shaded areas indicate the 2.5th-97.5th percentile range.

Before evaluating patients with dysfibrinogenemia, we established a normal reference range using 50 normal plasma samples (25 from healthy volunteers and 25 from the CRYOcheck Normal Donor Set). The α_2_AP levels in the normal plasma samples ranged from 0.84 to 1.30 IU/mL. Figure 4 shows the representative normal waveform with the 2.5th to 97.5th percentile range, and the corresponding parameter values are summarized in Table 1. The median (2.5th-97.5th percentile) *T*^lysis^ and *V*_max_^lysis^ was 173.6 seconds (141.3-208.1 seconds) and −0.175 (−0.274 to −0.134), respectively. The relationships between Fg level and various CLySis assay parameters in individual normal plasma samples were also investigated (Supplementary Figure). *V*_max_^clot^ correlated strongly with plasma Fg level (*r* = 0.9780), as previously described, and *V*_max_^lysis^ also showed a good negative correlation (*r* = −0.7121). By contrast, a negligible correlation was found with *T*^lysis^ (*r* = −0.2144), indicating that this parameter is largely independent of plasma Fg level. These results suggest that *T*^lysis^ (normal reference range, 141.3-208.1 seconds) is a more suitable stable reference, as it is not easily affected by Fg level per se.

**Table 1:** Normal reference ranges of CLySis assay parameters.

Parameter	Median (2.5th-97.5th percentile)
Fg:C (g/L)	2.70 (2.00-4.09)
α_2_AP (IU/mL)	1.11 (0.84-1.30)
*T*^clot^ (s)	12.7 (8.6-16.6)
*V*_max_^clot^	0.845 (0.484-1.886)
*T*^lysis^ (s)	173.6 (141.3-208.1)
*V*_max_^lysis^	−0.175 (−0.274 to −0.134)

α_2_AP, α_2_-antiplasmin; Fg:C, functional fibrinogen level, determined using Clauss fibrinogen assay.

**Figure 5:**
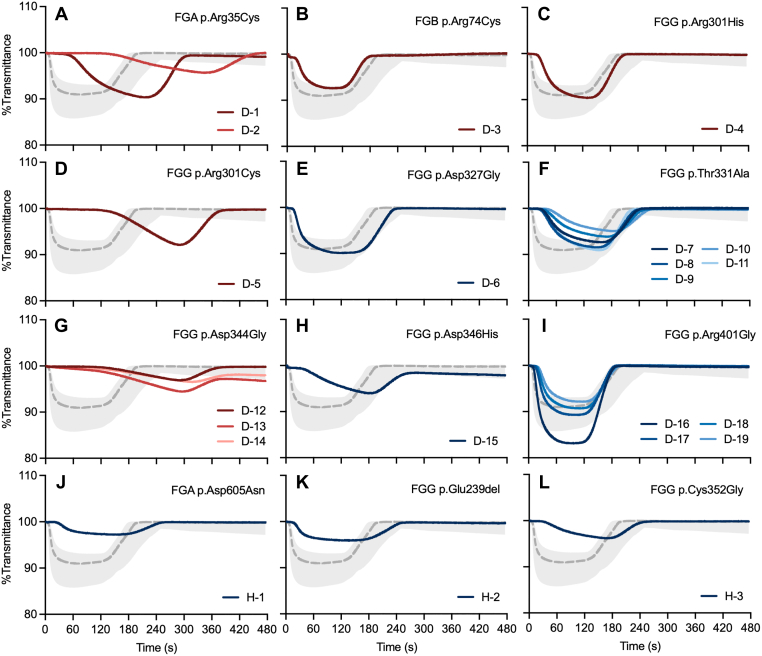
Clot-fibrinolysis waveforms obtained from the Clauss fibrinogen assay–based clot-fibrinolysis waveform analysis (CLySis) assay using plasma samples from patients with congenital fibrinogen disorders (CFDs). Waveforms are grouped according to the corresponding genomic variants. Gray dashed lines and shaded areas indicate the normal reference ranges. Red lines represent thrombosis-associated variants, whereas blue lines indicate variants not associated with thrombotic phenotypes. Each panel represents a distinct fibrinogen gene variant: (A) *FGA* p.Arg35Cys, (B) *FGB* p.Arg74Cys, (C) *FGG* p.Arg301His, (D) *FGG* p.Arg301Cys, (E) *FGG* p.Asp327Gly, (F) *FGG* p.Thr331Ala, (G) *FGG* p.Asp344Gly, (H) *FGG* p.Asp346His, (I) *FGG* p.Arg401Gly, (J) *FGA* p.Asp605Asn, (K) *FGG* p.Glu239del, and (L) *FGG* p.Cys352Gly.

We next investigated plasma samples from patients with CFDs (Figure 5). A total of 22 patients were enrolled, including 19 with congenital dysfibrinogenemia and 3 with congenital hypofibrinogenemia. Among the 19 patients with congenital dysfibrinogenemia, 10 distinct pathogenic variants were identified, with some patients carrying the same variant. We included several variants previously reported in patients with thrombosis in the literature, such as *FGA* p.Arg35Cys [3,5,18], *FGB* p.Arg74Cys [21], *FGG* p.Arg301His [3,5], *FGG* p.Arg301Cys [[3], [4], [5],^8^], and *FGG* p.Asp344Gly [5]. One of the patients carrying *FGG* p.Asp344Gly had a history of recurrent ischemic stroke, but the others did not, and no thrombotic or bleeding events were documented. Other variants—including *FGG* p.Thr331Ala [22], *FGG* p.Asp346His [8], and *FGG* p.Arg401Gly [23]—have not been reported as associated with thrombosis in previous studies, and no thrombotic events were documented in these patients. *FGG* p.Asp327Gly is a novel variant identified in a proband with postpartum hemorrhage. By contrast, the variants identified in patients with hypofibrinogenemia (*FGA* p.Asp605Asn, *FGG* p.Glu239del, and *FGG* p.Cys352Gly) were all novel, and all these patients had no thrombosis-related complications. Table 2 summarizes the characteristics of the pathogenic variants, laboratory findings, and corresponding CLySis assay results.

**Table 2:** Characteristics of patients with CFD and results of CLySis fibrinogen assays.

ID	Diagnosis and classification [1]	Pathogenic variant	Manifestation	Fg:C (g/L)	Fg:Ag (g/L)	Fg:C/Ag	α_2_AP (IU/mL)	*T*^clot^ (s)	*V*_max_^clot^	*T*^lysis^ (s)	*V*_max_^lysis^
D-1	Dysfibrinogenemia, 3A	*FGA* p.Arg35Cys	—	0.34	3.63	0.09	1.26	95.3	0.131	270.1	−0.189
D-2	Dysfibrinogenemia, 3A	*FGA* p.Arg35Cys	—	<30	2.34	<0.13	1.46	185.5	0.049	399.3	−0.070
D-3	Dysfibrinogenemia, 3B	*FGB* p.Arg74Cys	—	1.13	2.99	0.38	1.10	29.4	0.262	153.5	−0.185
D-4	Dysfibrinogenemia, 3A	*FGG* p.Arg301His	Pregnancy loss	0.80	2.17	0.37	1.13	41.4	0.263	179.0	−0.207
D-5	Dysfibrinogenemia, 3A	*FGG* p.Arg301Cys	—	<0.30	2.82	<0.11	1.12	207.4	0.085	349.4	−0.142
D-6	Dysfibrinogenemia, 3A	*FGG* p.Asp327Gly	PPH	1.17	3.09	0.38	1.15	28.5	0.395	206.4	−0.169
D-7	Dysfibrinogenemia, 3A	*FGG* p.Thr331Ala	—	0.65	1.78	0.37	1.16	50.5	0.162	221.8	−0.145
D-8	Dysfibrinogenemia, 3A	*FGG* p.Thr331Ala	—	0.67	2.63	0.25	1.27	48.7	0.183	204.2	−0.164
D-9	Dysfibrinogenemia, 3A	*FGG* p.Thr331Ala	—	0.55	1.59	0.35	1.17	59.0	0.107	220.8	−0.125
D-10	Dysfibrinogenemia, 4C	*FGG* p.Thr331Ala	Bruising	0.49	1.31	0.37	1.20	66.7	0.078	235.3	−0.099
D-11	Dysfibrinogenemia, 3A	*FGG* p.Thr331Ala	—	0.58	2.16	0.27	0.90	56.0	0.179	194.2	−0.210
D-12	Dysfibrinogenemia, 3A	*FGG* p.Asp344Gly	Ischemic stroke	<0.30	1.14	<0.26	1.10	146.0	0.043	345.5	−0.064
D-13	Dysfibrinogenemia, 3A	*FGG* p.Asp344Gly	—	0.34	1.48	0.23	1.20	93.9	0.060	341.5	−0.058
D-14	Dysfibrinogenemia, 3A	*FGG* p.Asp344Gly	—	<0.30	0.96	<0.31	1.15	110.1	0.031	360.7	−0.040
D-15	Dysfibrinogenemia, 4C	*FGG* p.Asp346His	—	0.78	1.28	0.61	1.46	42.1	0.116	216.6	−0.101
D-16	Dysfibrinogenemia, 3A	*FGG* p.Arg401Gly	PPH	1.62	4.16	0.39	1.44	21.0	0.880	152.8	−0.381
D-17	Dysfibrinogenemia, 3A	*FGG* p.Arg401Gly	—	1.22	2.61	0.47	1.02	27.3	0.467	152.5	−0.250
D-18	Dysfibrinogenemia, 3A	*FGG* p.Arg401Gly	—	1.07	2.26	0.47	1.05	31.1	0.378	160.4	−0.210
D-19	Dysfibrinogenemia, 3A	*FGG* p.Arg401Gly	—	0.96	1.97	0.49	1.10	34.6	0.276	163.2	−0.174
H-1	Hypofibrinogenemia, 2B	*FGA* p.Asp605Asn	Bruising	0.89	0.92	0.97	1.17	38.7	0.085	225.1	−0.055
H-2	Hypofibrinogenemia, 2C	*FGG* p.Glu239del	—	1.23	1.49	0.83	1.37	27.1	0.169	204.0	−0.087
H-3	Hypofibrinogenemia, 2B	*FGG* p.Cys352Gly	—	0.69	1.03	0.67	1.30	47.4	0.059	216.3	−0.077

α_2_AP, α_2_-antiplasmin; Fg:C, functional fibrinogen level, determined using Clauss fibrinogen assay; PPH, postpartum hemorrhage.

For *FGA* p.Arg35Cys (Figure 5A), *FGG* p.Arg301Cys (Figure 5D), and *FGG* p.Asp344Gly (Figure 5G), the CLySis assay showed significantly prolonged *T*^lysis^ compared with the normal reference range, reflecting altered clot formation–fibrinolysis kinetics, whereas both *FGB* p.Arg74Cys (Figure 5B) and *FGG* p.Arg301His (Figure 5C) were associated with normal *T*^lysis^ and *V*_max_^lysis^ values. Other variants also demonstrated normal *T*^lysis^ values, although *FGG* p.Asp346His showed a slightly prolonged T^lysis^ (216.6 seconds) (Figure 5H). The remaining variants—*FGG* p.Asp327Gly (Figure 5E), *FGG* p.Thr331Ala (Figure 5F), and *FGG* p.Arg401Gly (Figure 5I)—in patients with dysfibrinogenemia were almost within the normal reference range. Variants identified in patients with hypofibrinogenemia—*FGA* p.Asp605Asn (Figure 5J), *FGG* p.Glu239del (Figure 5K), and *FGG* p.Cys352Gly (Figure 5L)—also showed slightly prolonged *T*^lysis^ and lower *V*_max_^lysis^ values; however, the respective fibrinolysis waveforms overlapped entirely with those of the normal reference range.

### 3.4 Confirmation of altered clot formation–lysis kinetics using purified systems

**Figure 6:**
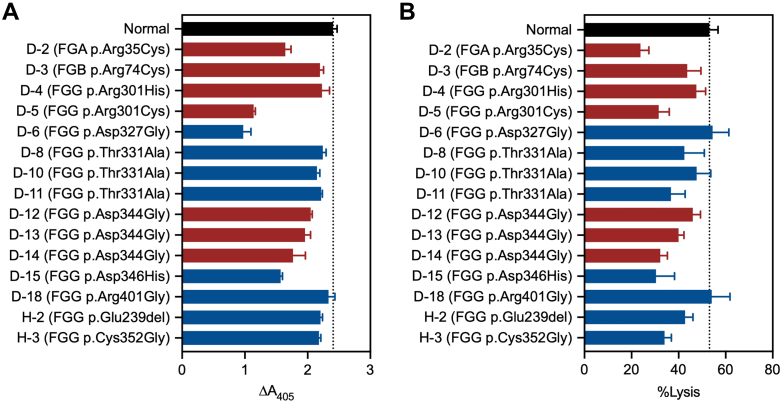
Plasmin generation assay and tissue-type plasminogen activator (tPA)-mediated fibrinolysis in purified dysfibrinogens. (A) Plasmin generation is indicated by the change in absorbance from baseline up to 90 minutes. (B) Percent lysis is shown as the relative change in absorbance at 150 minutes compared with that at time zero. Red bars represent known thrombosis-associated variants, and blue bars represent variants not associated with thrombotic phenotypes.

To further characterize the fibrin-dependent properties underlying the CLySis assay findings, dysfibrinogen was purified from patient plasma and analyzed using purified systems. PG (Figure 6A) and tPA-mediated clot lysis (Figure 6B) were evaluated using representative patient samples.

Dysfibrinogens with *FGA* p.Arg35Cys, *FGG* p.Arg301Cys, and *FGG* p.Asp344Gly were associated with reduced PG and impaired tPA-mediated clot lysis, whereas *FGB* p.Arg74Cys and *FGG* p.Arg301His were associated with nearly normal PG and clot lysis rates. *FGG* p.Asp327Gly exhibited reduced PG but a normal rate of tPA-mediated clot lysis. *FGG* p.Thr331Ala and *FGG* p.Arg401Gly were associated with almost normal PG and clot lysis rates. Unexpectedly, *FGG* p.Asp346His was associated with reduced PG and delayed clot lysis, similar to that observed in variants showing prolonged *T*^lysis^ in the CLySis assay. Among variants associated with hypofibrinogenemia, *FGA* p.Asp605Asn, *FGG* p.Glu239del, and *FGG* p.Cys352Gly showed normal PG; however, *FGG* p.Cys352Gly exhibited significantly delayed tPA-mediated clot lysis. Overall, the results of the PG and tPA-mediated clot lysis assays supported the CLySis assay findings, indicating that *T*^lysis^ reflects fibrin-dependent clot formation–lysis kinetics.

## 4 Discussion

Although phenotypic variation in congenital dysfibrinogenemia is well recognized, no conventional laboratory assays capable of characterizing dysfibrinogen properties or predicting clinical manifestations are available. Genotype analysis can provide useful information for interpreting phenotypes; however, genotypes are not always strongly correlated with clinical complications. The CLySis assay offers a novel, plasma-based approach for functional characterization of clot formation–lysis kinetics in dysfibrinogenemia and may serve as a useful exploratory tool to support future phenotypic analyses beyond conventional genotypic assessment.

In the present study, we established a novel exploratory assay to characterize integrated clot formation–lysis kinetics in dysfibrinogenemia. Conventional fibrinolysis assays, such as clot lysis time, would be valuable and could provide insights into fibrinolytic phenotypes. However, these plasma-based assays reflect the combined effects of multiple plasma components, including Pg, fibrinolysis inhibitors, and other cofactors, and are therefore not specifically designed to isolate Fg-dependent fibrinolytic properties. By contrast, a more specific system using purified Fg would be better suited to elucidate intrinsic fibrinolytic abnormalities; however, such systems are technically demanding and therefore not appropriate for an exploratory screening test. In this context, the Clauss Fg assay is widely used as an initial screening test, whereas the Clauss-CWA has been shown to be useful for classifying CFDs [14,15]. We developed the CLySis assay to further enhance the Clauss-CWA through simple supplementation with r-tPA and Lys-Pg. This modification enabled continuous monitoring of the fibrinolytic reaction following clot formation within the Clauss Fg assay framework, without interfering with the original clotting reaction. Lys-Pg was more effective than Glu-Pg at lower concentrations and therefore adopted in the CLySis assay protocol; however, the CLySis assay does not reflect the physiologic conversion of Glu-Pg to Lys-Pg. This may be a limitation when assessing the fibrinolytic potential induced by (dys)fibrinogen. Although the CLySis assay provided additional information related to fibrinolysis, the fibrinolytic parameters *T*^lysis^ and *V*_max_^lysis^ were affected by the plasma Fg level. The relationships between Fg concentration and these fibrinolytic parameters differed depending on the experimental system. Using purified Fg as the test sample, fibrinolysis occurred earlier as the Fg concentration decreased, indicating an increased susceptibility to fibrinolysis associated with lower Fg levels. By contrast, in the reconstituted plasma-based system, lower Fg concentrations (especially <1.0 g/L of Fg:Ag) resulted in a delay in *T*^lysis^, likely reflecting reduced incorporation of Pg into the fibrin network due to decreased (antigenic) fibrin formation. In addition, as reduced α_2_AP levels can offset the prolongation of *T*^lysis^, plasma α_2_AP activity should be measured and taken into account when interpreting CLySis results.

The CLySis assay provided functional information on fibrin-dependent clot formation–lysis kinetics that complemented genotypic data. In the present cohort, prolonged *T*^lysis^ was observed in several variants, including *FGA* p.Arg35Cys, *FGG* p.Arg301Cys, and *FGG* p.Asp344Gly, indicating altered clot–lysis behavior. These findings were supported by purified-system analyses, demonstrating reduced PG and delayed clot lysis. Unexpectedly, *FGB* p.Arg74Cys and *FGG* p.Arg301His were not associated with prolonged *T*^lysis^. *FGB* p.Arg74Cys, also known as Fg Nijmegen, is a well-documented variant, previously reported in patients with thrombosis and characterized by delayed tPA-mediated Pg activation and impaired tPA binding to dysfibrinogen, resulting in altered fibrin-dependent clot–lysis behavior [21]. In our purified system, PG and tPA-mediated clot lysis were slightly decreased compared with normal plasma; however, both *T*^lysis^ and *V*_max_^lysis^ were normal. Engesser et al. [21] demonstrated that Fg Nijmegen exhibits a partial impairment in tPA binding, with an ∼16% reduction compared with normal fibrin, whereas the binding of both Glu-Pg and Lys-Pg to fibrin has been reported to be comparable with that of normal fibrin. Accordingly, the primary defect of this variant is considered to be a relatively mild reduction in fibrin-bound tPA localization rather than a profound impairment of Pg activation. In the CLySis assay, the use of a relatively high concentration of tPA in combination with Lys-plasminogen likely compensates for such a modest reduction in tPA–fibrin interaction. Consequently, this subtle difference may fall below the detection sensitivity of the assay, resulting in *T*^lysis^ values within the normal range. We believe that this observation reflects an inherent limitation of the assay design, which is optimized to detect more pronounced altered clot–lysis behavior, rather than a contradiction of previous reports on Fg Nijmegen. Thus, prolonged *T*^lysis^ should be interpreted as a delayed transition from clot formation to fibrinolysis, reflecting integrated clot formation–lysis kinetics, rather than impaired initiation of fibrinolysis or fibrinolysis resistance per se.

Interestingly, the findings in *FGG* p.Arg301His variant in the present study differed significantly from those for the corresponding *FGG* p.Arg301Cys substitution. The Arg to Cys substitution at residue 301 of the Fg γ-chain is a nonconservative change that eliminates the strong positive charge of arginine and introduces a thiol group, likely causing a significant structural perturbation resulting in delayed fibrinolysis. By contrast, the Arg to His substitution partially retains the positive charge and represents a more conservative change, suggesting that the resulting structural and functional abnormalities are relatively mild [24]. This structural-chemical difference is consistent with our CLySis assays, in which *T*^lysis^ was prolonged with p.Arg301Cys but normal with p.Arg301His. Indeed, in the study by Mohsenian et al. [8], no patients carrying the *FGG* p.Arg301His variant experienced thrombotic events. These findings highlight the advantage of functional assays in differentiating fibrin-dependent clot formation–lysis behavior among dysfibrinogen variants.

Our study has several limitations. First, only a limited number of specific variants were examined, and major variants previously reported in patients with thrombosis such as *FGA* p.Arg573Cys (Fg Dusart) and *FGA* p.Ser551Cys (Fg Caracas V) [10] were not examined because we had no available patients carrying these variants. The performance of the CLySis assay should thus be validated through further large-scale studies. Second, the CLySis assay could not completely detect some variants previously reported in patients with thrombosis, such as *FGB* p.Arg74Cys (Fg Nijmegen). Prolonged *T*^lysis^ may associate with altered clot formation–lysis behavior of dysfibrinogen, but other mechanisms underlying fibrinolysis kinetics of dysfibrinogenemia cannot be evaluated using the current CLySis system. Additional approaches or further modifications will be required to clarify the molecular basis of these abnormalities. Third, many patients in our cohort who carried variants previously reported in patients with thrombosis were asymptomatic, consistent with previous reports [[6], [7], [8], [9]]. Although the CLySis assay results correlated well with the presence of certain variants showing altered clot formation–lysis kinetics, they did not directly correspond to the clinical symptoms. Therefore, delayed *T*^lysis^ should not be interpreted as direct evidence or prediction of past or future thrombosis but rather as a characteristic feature of the abnormal Fg molecule. Identifying potential confounding factors involved in thrombosis and integrating them into the overall evaluation will be essential for a comprehensive interpretation of the results.

In conclusion, we developed a novel Clauss-CWA–based assay for integrated assessment of clot formation and fibrinolysis kinetics in dysfibrinogenemia. The CLySis assay enables functional characterization of fibrin-dependent clot–lysis behavior under standardized conditions without interfering with conventional Clauss-CWA parameters. This approach may serve as a useful exploratory tool for phenotypic analyses of dysfibrinogenemia in future studies.
