# Supplementary material for: An exploratory plasma-based functional assay for phenotypic characterization of fibrinolysis in dysfibrinogenemia
Source: Res Pract Thromb Haemost. 2026 Mar 25;10(3):103426. doi: 10.1016/j.rpth.2026.103426 (PMC13100283; doi:10.1016/j.rpth.2026.103426)
Supplement: Supplementary Figure [file mmc1.pptx]

## Slide 1
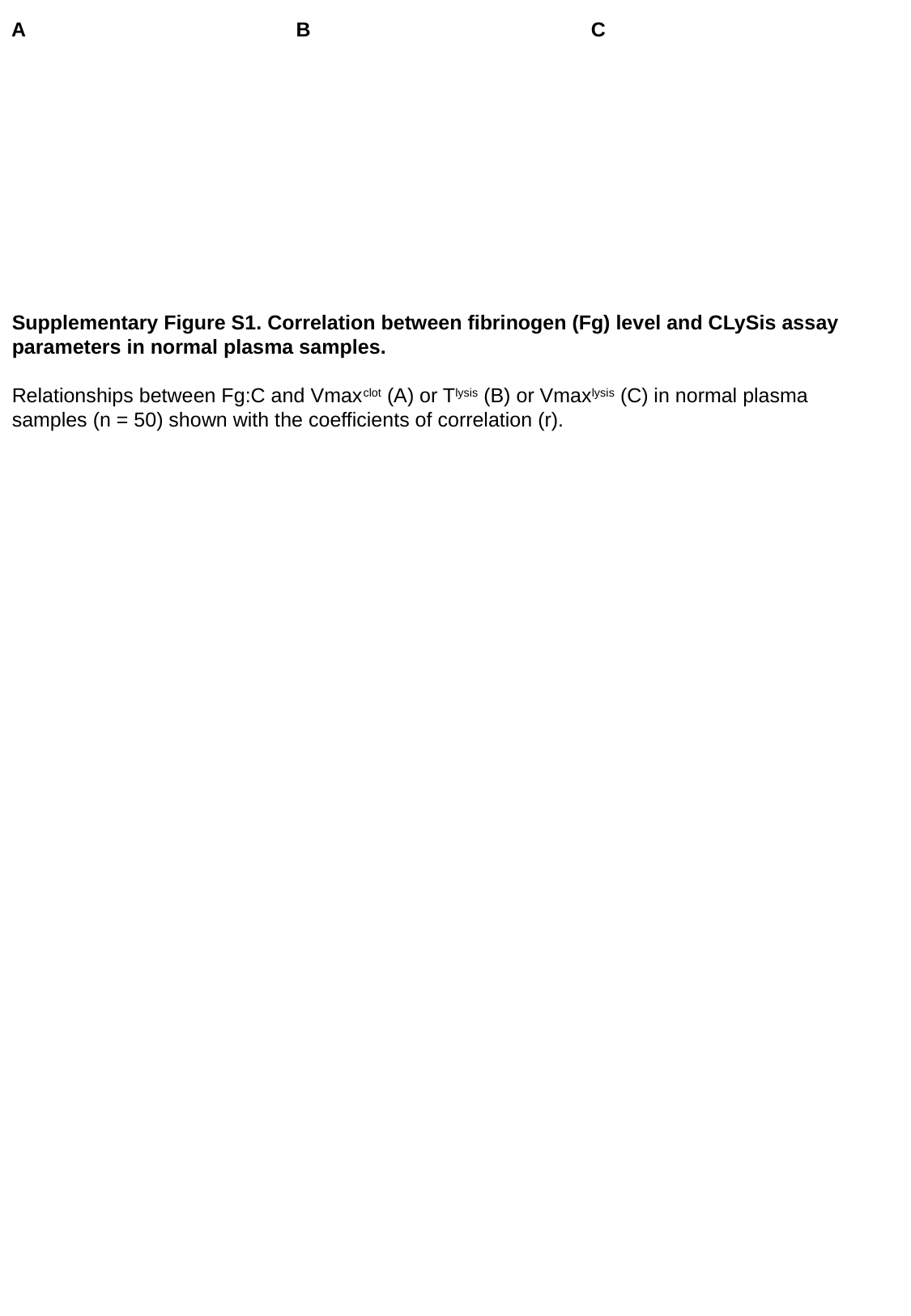

C
B
A
Supplementary Figure S1. Correlation between fibrinogen (Fg) level and CLySis assay parameters in normal plasma samples.
Relationships between Fg:C and Vmaxclot (A) or Tlysis (B) or Vmaxlysis (C) in normal plasma samples (n = 50) shown with the coefficients of correlation (r).

## Slide 2
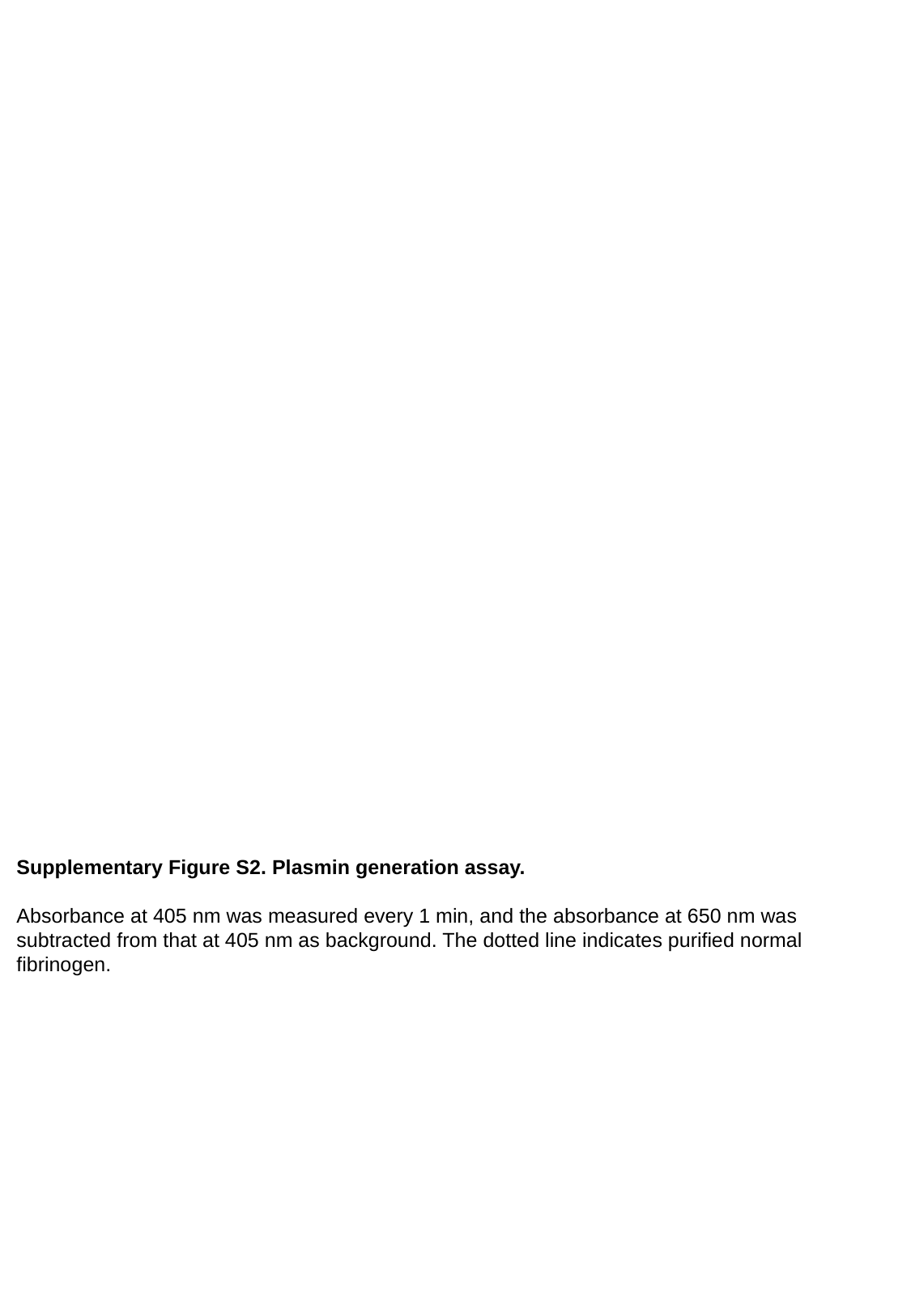

Supplementary Figure S2. Plasmin generation assay.
Absorbance at 405 nm was measured every 1 min, and the absorbance at 650 nm was subtracted from that at 405 nm as background. The dotted line indicates purified normal fibrinogen.

## Slide 3
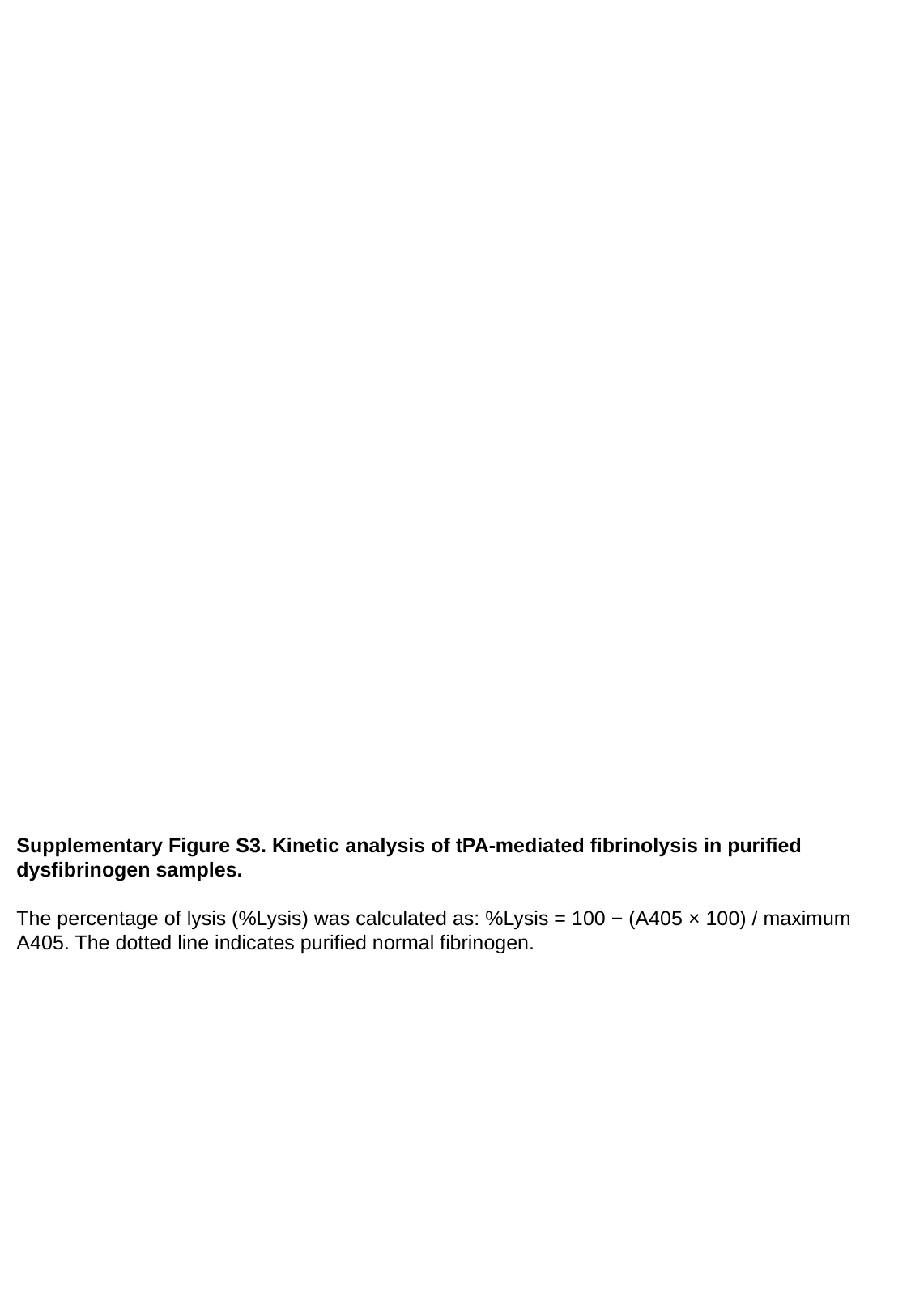

Supplementary Figure S3. Kinetic analysis of tPA-mediated fibrinolysis in purified dysfibrinogen samples.
The percentage of lysis (%Lysis) was calculated as: %Lysis = 100 − (A405 × 100) / maximum A405. The dotted line indicates purified normal fibrinogen.
