# Supplementary material for: An exploratory plasma-based functional assay for phenotypic characterization of fibrinolysis in dysfibrinogenemia
Source: Res Pract Thromb Haemost. 2026 Mar 25;10(3):103426. doi: 10.1016/j.rpth.2026.103426 (PMC13100283; doi:10.1016/j.rpth.2026.103426)
Supplement: Supplementary Tables 1-3 [file mmc2.docx]

**Supplementary Table S1. CLySis assay parameters for PgDP and PNP spiked with Glu- or Lys-Pg.**

|  |  | Pg Conc. (µM) |  |  |  |  |  |
| --- | --- | --- | --- | --- | --- | --- | --- |
|  |  | 0.2 | 0.1 | 0.05 | 0.02 | 0.01 | 0 |
| PgDP+Glu-Pg | T^clot^ | 12.6±0.1 | 12.6±0.1 | 12.6±0.1 | 12.6±0.2 | 12.5±0.1 | 12.5±0.2 |
|  | Vmax^clot^ | 0.828±0.005 | 0.816±0.011 | 0.816±0.013 | 0.818±0.014 | 0.825±0.004 | 0.825±0.003 |
|  | T^lysis^ | 337.7±9.3 | n/a | n/a | n/a | n/a | n/a |
|  | Vmax^lysis^ | -0.184±0.017 | n/a | n/a | n/a | n/a | n/a |
| PgDP+Lys-Pg | T^clot^ | 12.7±0.0 | 12.5±0.1 | 12.5±0.1 | 12.5±0.1 | 12.6±0.1 | 12.5±0.2 |
|  | Vmax^clot^ | 0.834±0.007 | 0.822±0.013 | 0.824±0.007 | 0.829±0.014 | 0.81±0.014 | 0.825±0.003 |
|  | T^lysis^ | 110.7±1.4 | 125.5±2.0 | 161.3±3.3 | n/a | n/a | n/a |
|  | Vmax^lysis^ | -0.302±0.008 | -0.214±0.009 | -0.118±0.003 | n/a | n/a | n/a |
| PNP+Glu-Pg | T^clot^ | 13.1±0.2 | 13±0.2 | 12.9±0.2 | 13.0±0.1 | 12.8±0.1 | 12.9±0.1 |
|  | Vmax^clot^ | 0.807±0.031 | 0.816±0.026 | 0.802±0.029 | 0.816±0.038 | 0.827±0.03 | 0.807±0.018 |
|  | T^lysis^ | 321.2±1.7 | 382.5±5.0 | 407.4±7.8 | 447.6±4.2 | 457.6±5.9 | 469.2±5.9 |
|  | Vmax^lysis^ | -0.174±0.004 | -0.17±0.012 | -0.16±0.019 | -0.146±0.007 | -0.143±0.008 | -0.143±0.005 |
| PNP+Lys-Pg | T^clot^ | 13.1±0.1 | 13.1±0.2 | 12.9±0.2 | 13.2±0.2 | 12.9±0.1 | 12.9±0.1 |
|  | Vmax^clot^ | 0.837±0.031 | 0.816±0.040 | 0.806±0.039 | 0.794±0.031 | 0.819±0.03 | 0.807±0.018 |
|  | T^lysis^ | 124.7±2.3 | 144.2±3.7 | 205.3±1.9 | 279.8±4.5 | 338.3±6.7 | 469.2±5.9 |
|  | Vmax^lysis^ | -0.243±0.007 | -0.185±0.007 | -0.154±0.007 | -0.145±0.008 | -0.148±0.008 | -0.143±0.005 |

Pg, plasminogen; PgDP, plasminogen-deficient plasma; n/a, not applicable

**Supplementary Table S2. CLySis assay parameters for plasminogen-depleted purified fibrinogen.**

| Fg (g/L) | T^clot^ | Vmax^clot^ | T^lysis^ | Vmax^lysis^ |
| --- | --- | --- | --- | --- |
| 4.5 | 7.7 | 0.758 | 155.8 | -0.131 |
| 3.6 | 8.7 | 0.529 | 134.1 | -0.126 |
| 2.7 | 10.7 | 0.306 | 121.1 | -0.114 |
| 1.8 | 13.9 | 0.141 | 119.3 | -0.09 |
| 0.9 | 22.8 | 0.047 | 123.7 | -0.064 |

**Supplementary Table S3. CLySis assay parameters for purified Fg-spiked Fg-deficient plasma**

| Fg (g/L) | T^clot^ | Vmax^clot^ | T^lysis^ | Vmax^lysis^ |
| --- | --- | --- | --- | --- |
| 5.70 | 6.8 | 0.703 | 168.4 | 0.131 |
| 3.88 | 9.7 | 0.286 | 155.1 | 0.100 |
| 2.80 | 13.2 | 0.127 | 159.9 | 0.074 |
| 2.36 | 15.0 | 0.077 | 176.0 | 0.059 |
| 1.34 | 25.5 | 0.042 | 197.8 | 0.045 |
| 0.79 | 40.7 | 0.035 | 226.7 | 0.035 |
